# A Novel Compressed Sensing Method for Magnetic Resonance Imaging: Exponential Wavelet Iterative Shrinkage-Thresholding Algorithm with Random Shift

**DOI:** 10.1155/2016/9416435

**Published:** 2016-03-15

**Authors:** Yudong Zhang, Jiquan Yang, Jianfei Yang, Aijun Liu, Ping Sun

**Affiliations:** ^1^Jiangsu Key Laboratory of 3D Printing Equipment and Manufacturing, Nanjing, Jiangsu 210048, China; ^2^Guangxi Key Laboratory of Manufacturing System & Advanced Manufacturing Technology, Guilin, Guangxi 541004, China; ^3^Department of Supply Chain Management, W. P. Carey School of Business, Arizona State University, P.O. Box 873406, Tempe, AZ 85287, USA; ^4^Department of Electrical Engineering, The City College of New York, CUNY, New York, NY 10031, USA

## Abstract

*Aim*. It can help improve the hospital throughput to accelerate magnetic resonance imaging (MRI) scanning. Patients will benefit from less waiting time.* Task*. In the last decade, various rapid MRI techniques on the basis of compressed sensing (CS) were proposed. However, both computation time and reconstruction quality of traditional CS-MRI did not meet the requirement of clinical use.* Method*. In this study, a novel method was proposed with the name of exponential wavelet iterative shrinkage-thresholding algorithm with random shift (abbreviated as EWISTARS). It is composed of three successful components: (i) exponential wavelet transform, (ii) iterative shrinkage-thresholding algorithm, and (iii) random shift.* Results*. Experimental results validated that, compared to state-of-the-art approaches, EWISTARS obtained the least mean absolute error, the least mean-squared error, and the highest peak signal-to-noise ratio.* Conclusion*. EWISTARS is superior to state-of-the-art approaches.

## 1. Introduction

Nyquist-Shannon Sampling Theorem (NSST) is a bridge connecting analogue signals and digital signals [1]. It says any signal can be completely reconstructed by a series of points spaced 1/(2*F*) seconds apart, when *F* represent the largest frequency of the signal, that is, the bandlimit [2]. Otherwise, the reconstruction is imperfect causing aliasing [3].

Magnetic resonance imaging (MRI) [4, 5] is prevalently used in both hospitals and institutes for neuroimaging of brains, compared to traditional X-ray, CT [6], and so forth. Technicians usually need to acquire full *k*-space data points, and the acquiring procedure is time-consuming. Hence, it is necessary to develop rapid MRI approach. In the last decade, compressed sensing (CS) was applied to accelerate MRI acquiring [7]. The compressed sensing magnetic resonance imaging (CS-MRI) consists of two main steps: random undersampling and image reconstruction. The former generates aliasing at random, and the latter removes the aliasing and recovers original image [8]. In this study, we focus on the latter.

For image reconstruction, Tikhonov regularization employed the *l*_2_-norm of undesirable residues and thus yields a closed-form linear solution [9]. Total Variation (TV) is only suitable for piecewise-constant patterns, since it usually selects finite difference as the sparsifying transform [10]. Afterwards, scholars proposed the iterative shrinkage/thresholding algorithm (ISTA) [11]. Nevertheless, it has a shortcoming of slow convergence speed.

In recent years, variants of ISTA were proposed. Subband adaptive ISTA (SISTA) seeks the optimal wavelet-subband related parameters [12]. Fast ISTA (FISTA) aims to speed up the convergence procedure [13]. SPGL1 is an efficient solver for large-scale one-norm regularized least squares, developed on the platform of Matlab [14]. NESTA is a robust and rapid first-order approach in order to solve basis-pursuit problems [15]. Fast composite splitting algorithm (FCSA) combines operator splitting and variable splitting [16]. C-SALSA is more general than SPGL1 in the sense that it can be used with any convex regularizer *φ* [17].

Our team focuses on developing more efficient ISTA based variants. In the past, we have already proposed to replace conventional wavelet transform (WT) with exponent of wavelet transform (EWT), which was a more efficient way for sparse representation than WT. We termed the method as exponential wavelet ISTA (EWISTA) [18]. Afterwards, we published a 2-page letter that proposed a rough concept, which embeds random shift (RS) technique to EWISTA, and named it as exponential wavelet ISTA with random shift (EWISTARS) [19]. In this study, we aim to purify the model, give mathematical support, and offer simulation results for the EWISTARS.

The rest of the paper is organized as follows: Section 2 offers the state-of-the-art progress on sparse representation and reconstruction algorithm.  Section 3 presents the proposed methodology.  Section 4 contains the experiment results and discussions. Finally, Section 6 is devoted to conclusion.

## 2. State of the Art

### 2.1. Sparse Representation

Discrete WT (DWT) is the most common sparsifying transform and is widely applied in a variety of academic and industrial fields [20–22]. In the last decade, scholars proposed various more efficient variants of DWT. Plonka [23] presented the easy path wavelet transform (EPWT). Khalidov et al. [24] proposed “activelets” to extract the activity-related component. Selesnick [25] studied the tunable Q-factor wavelet transform (TQWT) and proved it was suitable for sparsity-based inverse problems. Hao et al. [26] proposed to use “contourlets” as a new sparse transform in CS-MRI. Ning et al. [27] suggested to use patch-based directional wavelets (PBDW) that trained geometric directions from undersampled data. Qu et al. [28] designed a patch-based nonlocal operator (PANO), with the aim of sparsifying MR images. Huang et al. [29] developed a Bayesian nonparametric model for reconstructing MRIs based on severely undersampled data in *k*-space domain. Kayvanrad et al. [30] showed that penalizing the coefficients from translation-invariant stationary wavelet transform can reduce the visual pseudo-Gibbs artifacts. Srinivas et al. [31] proposed a sparsity model for histopathological image classification. Paquette et al. [32] found that the DWT with Cohen-Daubechies-Feauveau 9/7 wavelets, random radial sampling, and uniform angular sampling together gave excellent results. Pejoski et al. [33] used the discrete nonseparable shearlet transform (DNST) as a sparsifying transform and the FISTA for reconstruction. Fang et al. [34] took signals as a sparse linear combination in fractional Fourier transform domain. They treated the transform order as unknown. Li et al. [35] presented a dual-sparsity regularized sparse representation (DSRSR) model. Wang et al. [36] presented a fractional Fourier entropy technique.

Nevertheless, these algorithms were reported to cost a mass of computation resources. Our past work showed that exponential wavelet transform (EWT) simply calculates the exponent of WT and they can both increase the sparsity and reduce computation time [37]. Therefore, EWT was chosen as the sparse representation of this study.

### 2.2. Reconstruction Algorithm

Recently, scholars have found iterative algorithms can get better reconstruction for CS-MRI problem. Daubechies et al. [11] proposed the iterative shrinkage-thresholding algorithm (ISTA), which amounts to a Landweber iteration with thresholding applied every iteration step. Bioucas-Dias and Figueiredo [38] proposed a two-step IST (TwIST) algorithm. Beck and Teboulle [13] considered that ISTA converges quite slowly and presented a fast ISTA (FISTA). Bayram and Selesnick [12] investigated SISTA which is a subband adaptive version of ISTA. Guerquin-Kern et al. [39] proposed a variant of ISTA, which is the combination of recent improvements in convex optimization. Zhao et al. [40] reported an adaptively iterative shrinkage-thresholding (AIST) algorithm. Zhang et al. [37] proposed an algorithm called EWT-ISTA that combines both EWT and ISTA and demonstrated that their EWT-ISTA yielded fewer errors than ISTA. Balavoine et al. [41] studied the capacity of the standard ISTA to perform this task in real time. Konar et al. [42] proposed a Region of Interest Compressed Sensing (ROICS). Their method assumes that better performance is acquired when limiting the sparsity objective and data consistency in CS to a Region of Interest (ROI). Muckley et al. [43] proposed a B1-Based, Adaptive Restart, Iterative Soft Thresholding Algorithms (BARISTA). Yang et al. [44] presented a novel, two-stage reconstruction scheme for CS-MRI problem. Lingala et al. [45] proposed a new deformation corrected compressed sensing (DC-CS) method so as to recover undersampled MR images. Wei et al. [46] offered a novel approach for synthetic aperture radar tomography (TomoSAR) based on two-step iterative shrinkage/thresholding (TWIST). Liu and Lu [47] firstly transformed the problem of *l*_1_ norm data-fitting to *l*_1_ norm regularized *l*_2_ norm data-fitting. Secondly, they used FISTA to solve the equivalent problem. Hence, they proposed a rapid *l*_1_ linear estimation algorithm. Cauley et al. [48] proposed a hierarchically semiseparable (HSS) solver to represent the inverse of the CS+SENSE encoding matrix.

Let us revisit the above literatures; the variants of ISTA are now attracting more attention than traditional methods not only in the field of CS-MRI reconstruction but also in other applications. In this study, we would like to embed newly proposed concepts (the EWT and RS) into ISTA, in order to propose a novel and excellent CS-MRI reconstruction method.

## 3. Methodology

### 3.1. Reconstruction Model

Suppose *U* denotes the undersampling scheme in the *k*-space, namely, the incomplete Fourier transform. The data model of magnetic resonance imaging (MRI) scanner is written as

(1) $$\begin{array}{c} y=Ux+e. \end{array}$$

Here, *x* represents the original image, *y* is the measured data in *k*-space, and *e* is caused by either scanner imprecisions or the noise. Assume that *ω* denotes the sparsity coefficients; (1) can be transformed into the sparsity form as

(2) $$\begin{array}{c} y=Q\omega +e. \end{array}$$

Here, *Q* represents the system matrix that transforms from wavelet domain to *k*-space domain and *Q* = *UW* where *W* represents the inverse sparsifying transform. The reconstruction of *x* is transformed solving the following constrained optimization problem [49]:

(3) $$\begin{array}{c} \omega^{*}=\underset{\omega }{\arg\min}\;S\left(\;\omega \;\right), \end{array}$$

where *S* represents the cost function with definition of

(4) $$\begin{array}{c} S\left(\;\omega \;\right)={\left\|\;y-Q\omega \;\right\|}_{2}^{2}+\lambda {\left\|\;\omega \;\right\|}_{1}. \end{array}$$

Here, *λ* is a parameter controlling the fidelity degree of the reconstruction to the measurements.

### 3.2. Exponential Wavelet Transform

The wavelet transform (WT) belongs to one of the prevalent tools applied in compressed sensing magnetic resonance imaging (CS-MRI) [50]. The reason is the similarity between the brain texture characteristics and the wavelet functions [18]. The WT transformed a signal into wavelet domain. Since the coefficients in wavelet domain are usually sparse, WT is also treated as a sparsity transform with sparse representation given in the following:

(5) $$\begin{array}{c} T_{W}\left(\;x\;\right)=\omega , \end{array}$$

where *T*_*W*_ represents the wavelet transform (note that *T*_*W*_ = *W* ^−1^). Equation (5) reflects that *T*_*W*_ maps the spatial image *x* to the sparsity coefficients *ω*.

If the significant coefficients are enhanced and the small coefficients are suppressed, the sparsity transform will be enhanced. There are many nonlinear transforms, which can handle this problem. A latest solution is exponent wavelet transform (EWT) as

(6) $$\begin{array}{c} T_{E}\left(\;x,k\;\right)=T_{E}\left(\;T_{E}\left(\;x,1\;\right),1\;\right). \end{array}$$

Here, *k* is the number of exponential iterations and *T*_*E*_ is the exponential wavelet transform. A single (*k* = 1) EWT is implemented by

(7) $$\begin{array}{c} T_{E}\left(\;x,1\;\right)=\frac{\exp\left(T_{W}\left(x\right)\right)-1}{e-1}. \end{array}$$

In all, the procedures of the standard EWT contained three steps.

Pseudocode of exponential wavelet transform (EWT) is as follows:

*Step  1*. Input the randomly undersampled MRI signal or image. 

*Step  2*. Repeat *k* times.

*Step  2.1*. Carry out the wavelet transform (WT). 

*Step  2.2*. Normalize wavelet coefficients to [0  1]. 

*Step  3*. Output the EWT coefficients.

### 3.3. ISTA

The iterative shrinkage/thresholding algorithm (ISTA) presents a sequence of estimates *ω*_*n*_, which gradually approximates to the optimal result *ω* ^*∗*^. A temporary cost function *S*′ is defined with *ω*_*n*+1_ as the next estimate:

(8) ωn+1argminω ⁡S′ω,ωn=argminω ⁡Sω+ω,ωnΥ−QHQ2.

Note that (*Υ* − *Q* ^*H*^ *Q*) is positive definite. *Υ* serves as a tuning parameter. We can write the pseudocode of iterative shrinkage/thresholding algorithm (ISTA) as follows:

(9) $$\begin{array}{c} \omega_{n+1}⟵\Gamma_{2\lambda /J}\left(\;\omega_{n}+\frac{2}{J}\left(\;a-A\omega_{n}\;\right)\;\right), \end{array}$$

where

(10) a=QHy,

(11) A=QHQ,

(12) J≥2λmaxQHQ,

where Γ is the shrinkage operator and *b* is the threshold:

(13) $$\begin{array}{c} \Gamma_{b}\left(\;z\;\right)=\mathrm{sgn}\left(\;z\;\right)\left(\;\left|\;z\;\right|-\min\left(\;\frac{b}{2},\left|\;z\;\right|\;\right)\;\right). \end{array}$$

### 3.4. Random Shift

Discrete wavelet transform (DWT) is translation variant, which means that the DWT of a translation of a particular signal is not equal to the translation of its DWT. The reason lies in the fact that only even-indexed elements are used in the decimation of DWT [51]. Random shift (RS) is a possible solution to guarantee translation invariance to a moderate extent. The goal is accomplished by selecting randomly shifted indexed elements for each decomposition level [52].

### 3.5. EWISTARS

We proposed the EWISTARS method with pseudocode listed in Pseudocode 1. The EWISTARS is composed of three success components: the sparsity of exponential wavelet transform (EWT), the simplicity of iterative shrinkage/thresholding algorithm, and translation invariance of RS.

### 3.6. Evaluation

To evaluate the performance of the proposed method, we employed there measures: mean absolute error (abbreviated as MAE), mean-squared error (abbreviated as MSE), and peak signal-to-noise ratio (abbreviated as PSNR). Those indicators measure the reconstruction performance between the estimated image *x* ^*∗*^ and the original one *x* (see Table 1).

## 4. Results and Discussions

### 4.1. Algorithm Comparison

First, the proposed EWISTARS was compared with ISTA [11], SISTA [12], FISTA [13], and FCSA [16]. We use a partially collapsed vertebrae image and a brain image. Both images are of the same sizes of 256 × 256. For fair comparison, the coefficients normalization is implemented.

**Figure 1:**
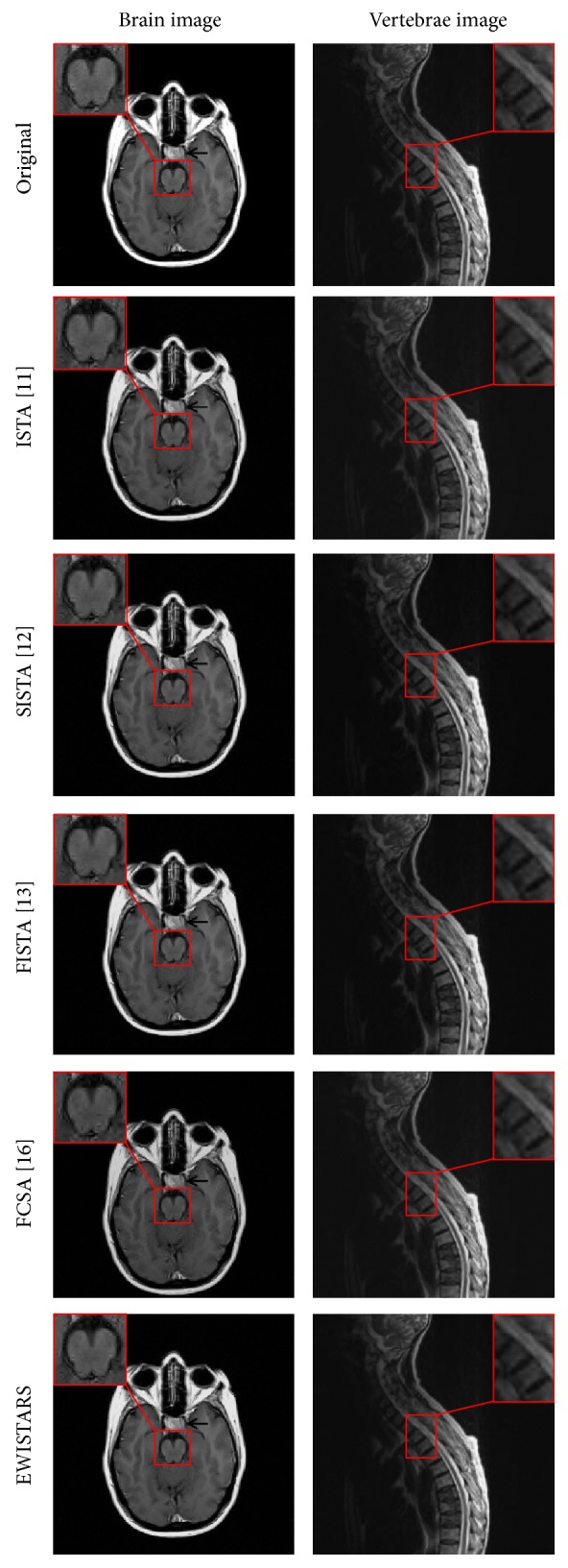
Comparison to state-of-the-art approaches.

The acceleration factor is assigned with a value of 5. Parameter *k* of EWISTARS is assigned with a value of 6 (see Section 4.3). We choose 5-level bior4.4 wavelet (see Section 4.4). Noise is inevitably contained in *k*-space. White Gaussian noise with standard deviation of 0.01 is mixed to the data points in *k*-space. The maximum iteration number was set to 100.  Figure 1 shows the comparison results. Detailed data of two images are listed in Tables 2 and 3.

### 4.2. Convergence Analysis

**Figure 2:**
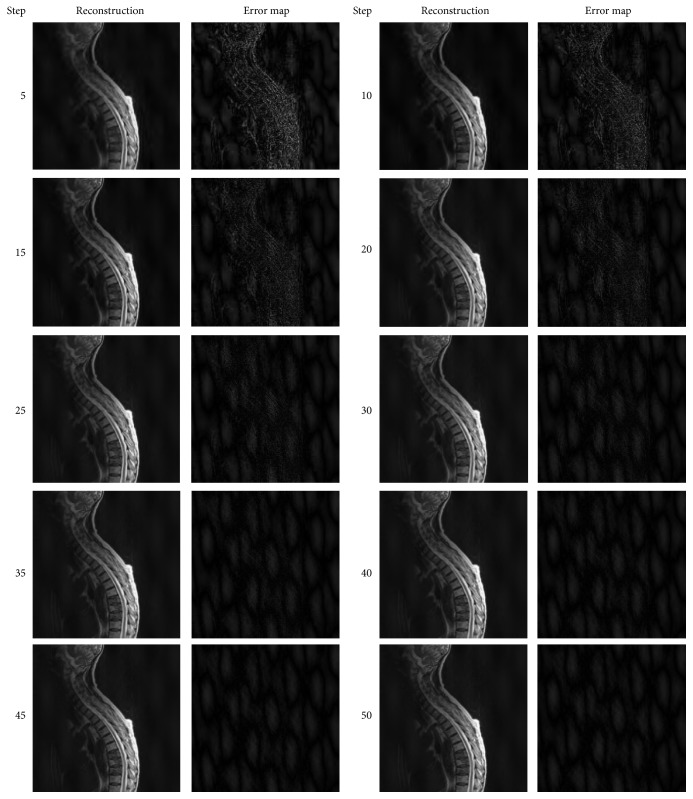
Convergence analysis of EWISTARS.

Here, we analyzed the convergence performance of EWISTARS over 50 steps. The error map was obtained by the difference between reconstruction image and original image. The error maps are brightened for better view. The results are shown in Figure 2.

### 4.3. Parameter Setting

**Figure 3:**
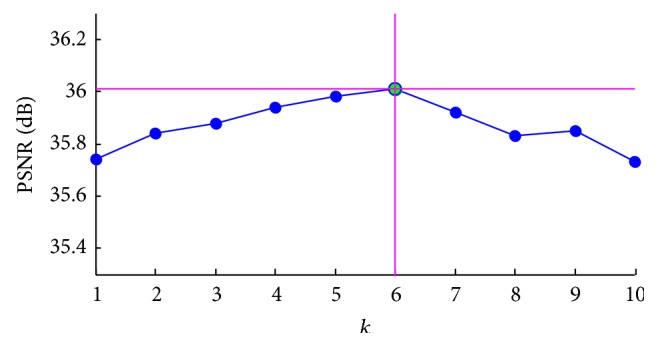
PSNR varies with *k* for 256 × 256 brain MR image.

How to optimize the parameter *k* in formula (6) remains a problem. We used the 256 × 256 brain MR image and changed the value of *k* from 1 to 10 with equal increment of 1.  Figure 3 shows the PSNR changes with *k*.

### 4.4. Optimal Wavelet Selection

In the fourth experiment, we compared six different wavelets on both images, in order to select the optimal wavelet. The 6 wavelets are introduced from both Daubechies family (db1, db2, and db3) and bior family (bior2.2, bior3.3, and bior4.4). Simulation setting is equal to that in Section 4.1.  Table 4 lists the corresponding PSNR results.

### 4.5. Explanation of Superiority of Bior4.4

**Figure 4:**
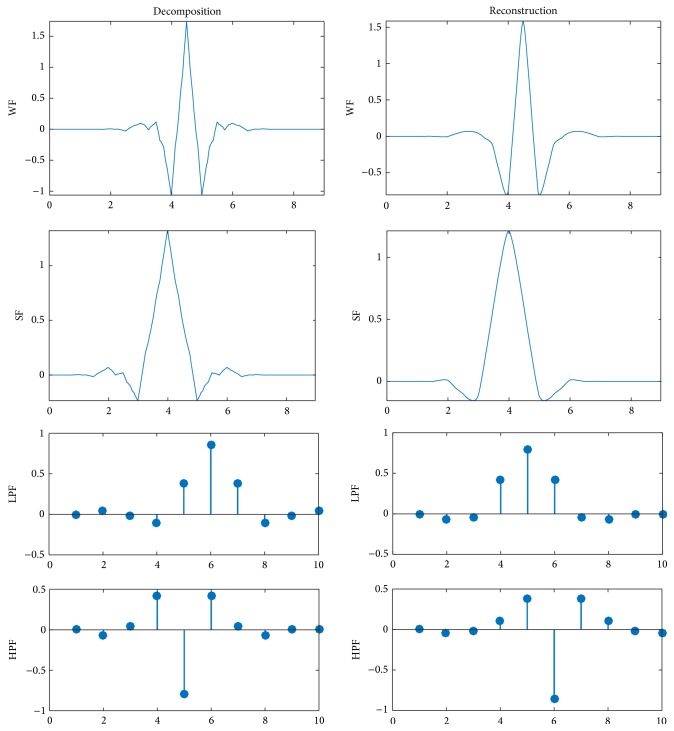
Analysis of bior4.4 wavelet.

To further explore the superiority of bior4.4 wavelet, Figure 4 draws its wavelet function (WF), scaling function (SF), low-pass filter (LPF), and high-pass filter (HPF) under two conditions: decomposition and reconstruction.

## 5. Discussions

Figure 1 shows the reconstruction results by five different methods over the vertebrae and brain images. Visually, those figures suggest that our EWISTARS yields more efficient performances than other approaches in suppressing noises and preserving brain tissues.

Table 2 offers the detailed evaluation of all algorithms over brain image. Our EWISTARS obtains the least MAE of 2.63, which is less than ISTA [11] of 2.72, SISTA [12] of 2.68, FISTA [13] of 2.67, and FCSA [16] of 3.50. The EWISTARS obtains the least MSE of 16.31, compared to ISTA [11] of 17.74, SISTA [12] of 16.90, FISTA [13] of 16.98, and FCSA [16] of 40.66. Besides, the EWISTARS obtains the largest PSNR of 36.01 dB, higher than ISTA [11] of 35.64 dB, SISTA [12] of 35.85 dB, FISTA [13] of 35.83 dB, and FCSA [16] of 32.04 dB. All those three measures indicate the superiority of EWISTARS in terms of reconstruction. For the computation time, FCSA expenses the least time of 4.66 seconds, ISTA [11] costs 10.47 seconds, SISTA [12] costs 8.23 seconds, FISTA [13] costs 8.49 seconds, and our EWISTARS costs 9.43 seconds.

Table 3 gives quantified results of all algorithms over vertebrae image. The ISTA [11] obtains MAE of 1.43, MSE of 7.16, and PSNR of 39.58 dB and costs 9.57 seconds. SISTA [12] obtains MAE of 1.37, MSE of 5.91, and PSNR of 40.42 dB and costs 7.19 seconds. FISTA [13] obtains MAE of 1.38, MSE of 6.22, and PSNR of 40.19 dB and costs 7.40 seconds. FCSA [16] obtains MAE of 1.49, MSE of 8.38, and PSNR of 38.90 dB and costs 5.17 seconds. Finally, the proposed EWISTARS obtains MAE of 1.30, MSE of 4.92, and PSNR of 41.21 dB and costs 7.68 seconds. In summary, the EWISTARS again shows better reconstruction quality than other four algorithms.

Why does the proposed EWISTARS success? The reason may lie in three points. First, the exponential wavelet transform gives enhanced sparsifying transform than other sparse representations. Second, our model inherits the simplicity and rapidness of traditional iterative shrinkage-thresholding algorithm. Finally and most importantly, the introduced random shift technique alleviates the translational variance of discrete wavelet transform used in state-of-the-art approaches. All those three reasons help to enhance the performance of our EWISTARS.

We can find in Figure 3 that the optimal value of *k* is 6, since it corresponds to the highest PSNR. As we discussed, increase of *k* from 0 leads to the sparsity enhancement; hence, the PSNR will also increase. However, calculation error accumulates when *k* is too large (*k* is greater than 5 in this situation). Therefore, 6 may be the most appropriate value of *k*. For the vertebrae image, the result is the same.

PSNRs in Table 4 show that db1 wavelet yields 34.49 decibels (dB) for brain image and 38.63 dB for vertebrae image, which is the worst among all wavelets. The db2 wavelet yields 35.41 dB and 40.82 dB for brain and vertebrae images, respectively. The db3 yields 35.40 dB and 40.89 dB for brain and vertebrae images, respectively. For the bior family, the bior 2.2 yields 35.79 dB and 40.76 dB for brain and vertebrae images, respectively. The bior3.3 yields 34.88 dB and 39.94 dB for brain and vertebrae images, respectively. Finally, the bior4.4 yields 36.01 dB and 41.21 dB for brain and vertebrae images, which are the highest PSNR values.

In Figure 4, we find that WF and SF of bior4.4 are similar to gray-level texture changes of the human tissues. Those texture changes are abundant in either human brains or vertebrae parts. Therefore, bior4.4 is more effective than other wavelets.

## 6. Conclusion and Future Research

In this study, a novel EWISTARS algorithm was proposed. Experiments validated its superiority to state-of-the-art techniques. Our contribution is twofold: (i) we presented a purified mathematical model for EWISTARS and gave its fast reconstruction algorithm and (ii) we tested its superiority to state-of-the-art approaches with regard to three different measures.

Future work is to test and include more efficient sparsifying transform and more efficient ISTA variants, to improve the effectiveness and efficiency of reconstruction of CS-MRI. Meanwhile, this proposed EWISTARS method may be used in combination with other postprocessing techniques, such as classification [53], detection [54], and recognition [55]. Privacy [56] is another topic to be researched during scanning.

## Figures and Tables

**Pseudocode 1:**
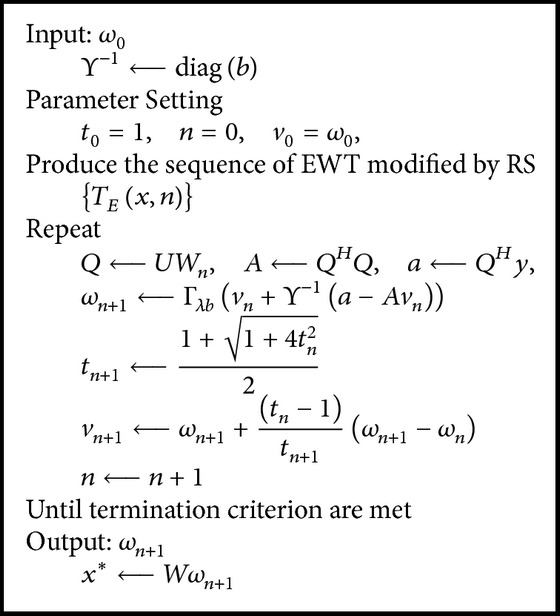
Pseudocode of EWISTARS.

**Table 1:** Definitions of reconstruction indicators (*x* ^*∗*^ represents the optimal estimate of original image *x*).

Indicator	Abbreviation	Definition
Mean-squared error	MSE	$\frac{1}{N}\overset{N}{\underset{i=1}{\sum }}{\left(x\left(i\right)-x^{*}\left(i\right)\right)}^{2}$
Mean absolute error	MAE	$\frac{1}{N}\overset{N}{\underset{i=1}{\sum }}\left|x\left(i\right)-x^{*}\left(i\right)\right|$
Peak signal-to-noise ratio	PSNR	$20\;\log_{10}\left(\frac{255}{\sqrt{MSE}}\right)$

**Table 2:** CS-MRI algorithm comparison over brain image (bold means the best).

	MAE	MSE	PSNR	Time
ISTA [11]	2.72	17.74	35.64	10.47
SISTA [12]	2.68	16.90	35.85	8.23
FISTA [13]	2.67	16.98	35.83	8.49
FCSA [16]	3.50	40.66	32.04	**4.66**
EWISTARS (proposed)	**2.63**	**16.31**	**36.01**	9.43

PSNR is in unit of dB and time is in unit of second.

**Table 3:** CS-MRI algorithm comparison over vertebrae image (bold means the best).

	MAE	MSE	PSNR	Time (s)
ISTA [11]	1.43	7.16	39.58	9.57
SISTA [12]	1.37	5.91	40.42	7.19
FISTA [13]	1.38	6.22	40.19	7.40
FCSA [16]	1.49	8.38	38.90	**5.17**
EWISTARS (proposed)	**1.30**	**4.92**	**41.21**	7.68

PSNR is in unit of dB and time is in unit of second.

**Table 4:** PSNR values of different wavelets (bold represents the best).

Wavelet	Brain	Vertebrae
db1	34.49	38.63
db2	35.41	40.82
db3	35.40	40.89
bior2.2	35.79	40.76
bior3.3	34.88	39.94
bior4.4	**36.01**	**41.21**
